# Knee Injury and Osteoarthritis Outcome Score (KOOS) Responder Criteria and Minimal Detectable Change 3–12 Years Following a Youth Sport-Related Knee Injury

**DOI:** 10.3390/jcm10030522

**Published:** 2021-02-01

**Authors:** Clodagh M. Toomey, Jackie L. Whittaker, Luz Palacios-Derflingher, Carolyn A. Emery

**Affiliations:** 1 Sport Injury Prevention Research Centre, Faculty of Kinesiology, University of Calgary, Calgary, AB T2N 1N4, Canada; jackie.whittaker@ubc.ca (J.L.W.); lmpalaci@ucalgary.ca (L.P.-D.); caemery@ucalgary.ca (C.A.E.); 2 School of Allied Health, Faculty of Education and Health Sciences, University of Limerick, V94 T9PX Limerick, Ireland; 3 McCaig Institute for Bone and Joint Health, University of Calgary, Calgary, AB T2N 4Z6, Canada; 4 Alberta Children’s Hospital Research Institute, University of Calgary, Calgary, AB T2N 4N1, Canada; 5 Department of Physical Therapy, Faculty of Medicine, University of British Columbia, Vancouver, BC V6T 1Z3, Canada; 6 Arthritis Research Canada, Richmond, BC V6X 2C7, Canada; 7 Department of Community Health Sciences, Cumming School of Medicine, University of Calgary, Calgary, AB T2N 4N1, Canada; 8 Department of Pediatrics, Cumming School of Medicine, University of Calgary, Calgary, AB T2N 4N1, Canada

**Keywords:** knee, ACL, injury, osteoarthritis, KOOS, musculoskeletal disorders, symptoms, pain

## Abstract

The applicability of thresholds that constitute an acceptable score or meaningful change on the Knee injury and Osteoarthritis Outcome Score (KOOS) in cohorts ≥ 5 years following knee injury is not well understood. The primary objective of this study was to evaluate the association between intra-articular knee injury type and two different KOOS pain thresholds (patient acceptable symptom state (PASS) and Englund symptomatic knee criteria) in the Alberta Youth Prevention of Osteoarthritis (PrE-OA) cohort, which includes participants 3–12 years following a youth sport-related knee injury and uninjured controls with similar age, sex and sport characteristics. Analyses accounted for sex, time since injury and the interaction between time since injury and injury type. Secondary objectives were to report proportions meeting thresholds for KOOS outcomes and minimal detectable change (MDC) from published test–retest reliability data, over a 1–4-year follow-up. Two hundred and fifty-three (253) participants (124 injured, 129 controls) were included in analyses, of which 153 (77 injured, 76 controls) had follow-up data. Similar odds were observed for presence of pain (below PASS threshold) in participants with anterior cruciate ligament (ACL)/meniscus injury (odds ratio (OR) 4.2 (97.5% confidence interval (CI): 1.8, 9.9)) and other knee injuries (OR 4.9 (97.5% CI: 1.2, 21.0)), while there were higher odds for presence of Englund “symptomatic knee” criteria in participants with ACL/meniscus injury (OR 13.6 (97.5% CI: 2.9, 63.4)) than other knee injuries (OR 7.3 (97.5% CI: 0.8, 63.7)) compared to controls. After a median 23.4 (8 to 42) month follow-up, 35% of previously injured participants had at least one KOOS sub-scale score that worsened by more than the MDC published threshold. Despite limited research, this study shows that individuals with youth sport knee injuries other than ACL or meniscus injury may also experience significant pain and symptoms 3–12 years following injury. Replication and further follow-up are needed to identify a possible clinical trajectory towards osteoarthritis.

## 1. Introduction

Much sport medicine and musculoskeletal health research has focused on rehabilitation and recovery following an anterior cruciate ligament (ACL) injury due to the immediate and long-term effects on activity participation and health. The negative consequences on health outcomes that are present in this population in the short-term (1–2 years) [1,2] and medium-term (3–12 years) [3,4,5] interval following injury, implicate knee injuries as one of the largest public health injury-related burdens. Of foremost concern is the higher likelihood of development of knee joint osteoarthritis (OA) after sustaining an ACL or other traumatic intra-articular knee injury [6], sentencing a proportion of these individuals to a lifetime of knee pain, symptoms, reduced function and quality of life (QOL). Of these patient-reported outcomes (PRO), knee pain is the most common symptom of OA [7], but defining what constitutes clinically significant pain is not well-understood in individuals following injury and prior to onset of potential joint disease.

The Knee injury and Osteoarthritis Outcome Score (KOOS) is a common PRO used to track the most prevalent clinical features across the timeline from knee injury to OA [8,9]. An alternative and more clinically interpretable method of observing KOOS has been to use defined thresholds of “patient acceptable symptom state” (PASS) in cohorts up to 5 years post-injury to understand what is deemed “acceptable” to a patient on each 0–100 sub-scale. Ingelsrud et al. [10] used anchor-based questions of a current satisfactory state regarding knee function in the Norwegian Knee Ligament Registry (NKLR) and took mean scores for KOOS subscales, where ‘yes’ was answered. Muller et al. [11] used a similar method in an ACL cohort 1–5 years from injury using the receiver operating characteristic method. Reports of PASS in these ACL cohorts construe that symptoms tend to stabilize and do not change much from 1 to 2 years postoperatively. Other methods to identify KOOS thresholds have used a consensus expert panel to define patients with a symptomatic knee, significant enough to seek medical attention, as applied by Englund et al. [12] to a cohort 16 years post-meniscectomy. Examining these thresholds in the medium-term interval following injury may provide an indication of the trajectory of PRO scores in a population at risk of OA. In addition, knowledge of outcomes in knee injuries other than ACL or meniscus injury is currently lacking.

The interpretation of longitudinal changes in PROs in the years following a knee injury is critical to identify individuals who may be on a trajectory to post-traumatic OA. This can be achieved using the minimal detectable change (MDC), to judge change that surpasses the instrument test–retest reliability [13]. These values may help to identify patients whose scores worsen over time and require an early post-traumatic OA intervention. It may also be helpful for clinicians to understand the baseline variables of other physiological (body composition) and performance (knee strength and function) outcomes in those who worsen over time, as this may aid with identification of at-risk individuals and mechanisms to target during intervention. This type of analysis has not previously been applied to longitudinal data of cohorts with different types of knee injuries.

The primary objective of this cohort study was to evaluate the association of intra-articular knee injury history type (uninjured, ACL and/or meniscal injury or other knee injury) with two types of KOOS criteria to define a painful or symptomatic knee, adjusting for time since injury and sex in a cohort 3–12 years after injury. Secondary objectives were to report the prevalence of acceptable or non-symptomatic PRO’s using defined KOOS thresholds and comparing all injury types in this cohort. An additional secondary objective was to evaluate the MDC in KOOS outcomes over a 1–4-year longitudinal follow-up, describing the clinical features and functional performance of injured participants who worsened on at least one sub-scale over time.

## 2. Materials and Methods

### 2.1. Participants and Recruitment

Participants include the entire cohort of the Alberta Youth Prevention of Early Osteoarthritis (PrE-OA) study, a historical longitudinal cohort study carried out at the Sport Injury Prevention Research Centre, Faculty of Kinesiology, University of Calgary. The PrE-OA cohort consists of youth and young adults who sustained an intra-articular knee injury 3–12 years prior to study recruitment, while participating in sport under the age of 18 years. Uninjured controls of similar age (≤1 year), sex and sport (at the time of injury) were recruited to match the injured participants. Detailed recruitment procedures have been described previously [4,14]. Knee injury was defined as a clinical diagnosis of knee ligament, meniscal, or other intra-articular tibiofemoral or patellofemoral injury that occurred playing sport and required both medical consultation and disrupted regular sport participation. Uninjured participants reported no previous time-loss knee injury. Participant exclusion criteria included pregnancy, non-steroidal anti-inflammatory use or cortisone injection within three months prior to testing, a musculoskeletal injury within the previous three months prior to testing that resulted in time loss (work, school or sport), diagnosis of other arthritides, or any current medical problem that prevented participation in the functional testing aspect of the study (e.g., neurological conditions). Participants were recruited between 2013 and 2017 for their initial visit and were invited to return for repeat testing annually for two subsequent visits. If a participant withdrew from the study before completion, every effort was made to replace that participant with an individual with the same characteristics of age (≤1 year), sex and sport at the time of injury. Participants were no longer considered ‘uninjured’ if they sustained a knee injury in the follow-up period. All tests were performed on the same day with random order of stations and rests between tests to minimize fatigue. Ethics approval was granted from the Conjoint Health Research Ethics Board at the University of Calgary, Canada (REB-14-2212), and all participants provided signed informed consent or assent where applicable.

### 2.2. Outcome Measures

Participants completed a study questionnaire that gathered demographic details, sport participation and medical, injury and surgery history detail. The Knee Injury and Osteoarthritis Outcome Score (KOOS) survey was completed, which provided information on knee function related to five sub-scales: pain, other symptoms, activities of daily living (ADL), sport and recreation and quality of life (QOL). The KOOS has been validated in knee injury populations and has high test–retest reliability [9], with higher scores indicating better outcomes. Two criteria were applied to the KOOS scores to determine the proportion of participants meeting an acceptable threshold (PASS) [10,11] for each subscale and KOOS4 [10,15] (average KOOS score of all subscales except KOOS ADL). Additional criteria were applied to identify a “symptomatic knee” based on Englund et al.’s [12] thresholds—defined by having QOL and at least two other subscales below the cut-off. For longitudinal assessment, minimal detectable change (MDC) criteria were used to observe change that surpassed the test–retest reliability of KOOS. As a conservative approach, the upper limits reported by Collins et al. [13] were used. Thresholds for each criterion are presented in Table 1.

As reported in previous publications [4,14], participants’ knee extensor strength, hop performance and body composition were measured. Normalized knee extensor isometric strength was assessed using handheld isometric dynamometry (model 01163; Lafayette Instrument, Lafayette, IN, USA) as described previously [3]. The peak isometric strength (N) scores were converted to torque values (N/m; force distance between joint line and dynamometer position) and normalized to body weight (N/m/kg^−^^1^). The maximum distance across two trials of the Triple Single Leg Hop (TLSH) test [3] was recorded for each leg and expressed as a percentage of leg length. Total body adiposity was assessed using dual-energy X-ray absorptiometry (DXA; Hologic Discovery (Hologic, Inc., Marlborough, MA, USA)). Whole-body fat mass (kg) was divided by height (m) squared to derive a fat mass index (FMI; kg/m^2^), to allow an accurate representation of adiposity that is not confounded by the presence of lean mass.

### 2.3. Statistical Analyses

All data were managed in REDCap (Research Electronic Data Capture) and statistical analyses were completed using Stata Version 15.1 (StataCorp LP, College Station, TX, USA). All outcomes were described using median with interquartile range (IQR) by injury type. Control participants were excluded from the analysis if there was no injured case with matching age, sex and sport criteria. For the primary objective and cross-sectional analysis, outcomes collected at the final measurement (furthest time-point from injury) were included due to a greater likelihood of observing changes related to post-traumatic OA. Multivariable logistic regression clustered on matched characteristics of sex and sport, and adjusted for time since exposure, sex and the interaction between injury type and time since exposure, was used to evaluate the odds (odds ratio (OR) and 97.5% confidence interval (CI)) of significant pain (Model 1) or a symptomatic knee (Model 2) by knee injury type (no knee injury history, grade III ACL tear and/or meniscal injury history, other knee injury history). Bonferroni adjustment was applied to CIs since there were two primary outcomes (α = 0.025). The outcome for Model 1 was the KOOS pain PASS criteria consistent from both Ingelsrud et al. [10] and Muller et al. [11] due to a high prevalence of pain in symptomatic OA. The outcome for Model 2 was the Englund et al. [12] criteria for a symptomatic knee. For the control participants, time since exposure was coded the same as that of the matched injured participants on recruitment corresponding to an equivalent injury-free time. Age was not included in the models due to collinearity with time since exposure. A backwards-stepwise elimination approach was employed, and the most parsimonious models reported using likelihood ratio tests and checking for a 10% difference in beta coefficients. For longitudinal analyses, the change in KOOS scores from the first and final follow-up for all participants was calculated and the frequency and proportions of individuals whose KOOS scores changed greater than MDC in either direction were reported. Baseline clinical features of injured participants who had ≥1 KOOS outcome that worsened over time using MDC were described alongside those who improved or stayed the same using median and IQR, or frequencies and proportions.

## 3. Results

**Figure 1:**
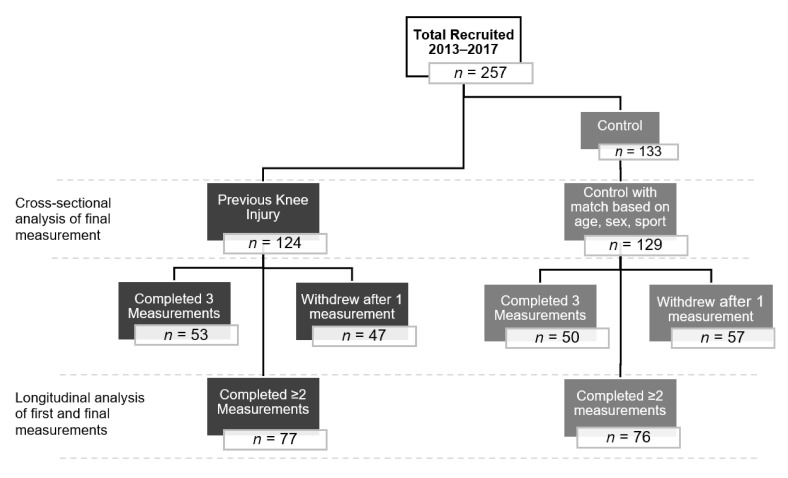
Flowchart of participant recruitment and follow-up for the Alberta Youth Prevention of Early Osteoarthritis (PrE-OA) cohort.

The final dataset was comprised of 253 participants (124 previously injured participants (*n* = 89 ACL and/or meniscal injury and *n* = 35 other knee injury) and 129 uninjured controls). A flowchart of participant recruitment and follow-up for these analyses is shown in Figure 1. Females comprised 55% of the sample and the median participant age was 24.2 years (range 15–30), median body mass index (BMI) 24.4 kg/m^2^ (range 18.1–38.9) at the furthest time-point from exposure. The median age of previously injured participants was 15.4 years (range 9–18 years) at the time of injury and 7.8 years (range 3–12.6 years) post-injury at the time of data collection. Injury type comprised mainly of third-degree ACL sprain (*n* = 69; 56%), all of which underwent surgical reconstruction. Of these, 51 (74%) also had an associated meniscal lesion. A further 16% (*n* = 20) of the total injured participants had a meniscal injury which did not include a third-degree ACL tear (*n* = 17 isolated meniscal injury and *n* = 3 associated with another injury). Eight of these underwent arthroscopic surgery. The ‘Other Injury’ category included other ligamentous injuries (first- to third-degree medial and lateral collateral ligament sprain, or first- to second-degree ACL or posterior collateral ligament sprain without meniscal involvement) (*n* = 15; 12%), patellar dislocations and subluxations (*n* = 18; 15%) and intra-articular fracture (*n* = 2). Injuries were sustained while playing 14 different sports: the most common sports were soccer (50%), basketball (18%) and ski/snowboarding (9%) for females, and ice hockey (42%), soccer (18%) and football (11%) for males. Participants were grouped into 21 clusters based on sex and sport, ranging between 2 and 69 participants per cluster.

Table 2 displays descriptive statistics for KOOS outcomes by injury type at the furthest time-point from injury, and the proportion of participants meeting thresholds by each criterion. For participants with an ACL and/or meniscal injury, the odds of not achieving an “acceptable” KOOS pain PASS score (Model 1) were 4.2 times (97.5% CI: 1.8, 9.9) the odds for uninjured controls, while those with other knee injuries had 4.9 times (97.5% CI: 1.2, 21.0) the odds of uninjured controls. Sex, time since exposure or interaction terms did not significantly influence these findings. In Model 2, the odds of meeting criteria for a symptomatic knee in those with an ACL and/or meniscus injury were 13.6 times (97.5% CI: 2.9, 63.4) the odds of uninjured controls, while those with other knee injuries had 7.3 times (97.5% CI: 0.8, 63.7) the odds of uninjured controls. Including sex as a covariate improved the likelihood ratio and r^2^ of the model but was not a significant predictor (OR 0.5 (97.5% CI: 0.2, 1.2)). Time since exposure or interaction terms did not influence Model 2.

**Figure 2:**
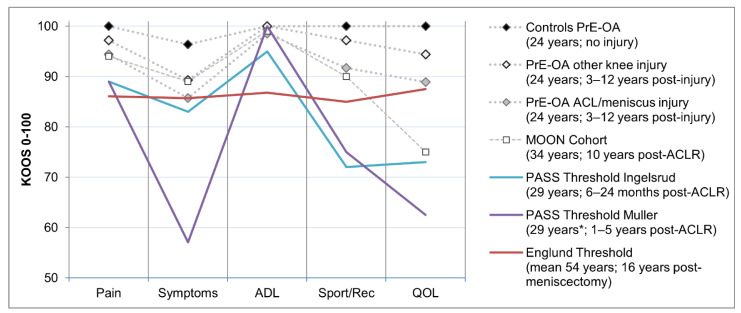
Median of KOOS subscales by injury type in the PrE-OA cohort compared to the Multicenter Orthopedic Outcomes Network (MOON) cohort and KOOS thresholds with median age and time since injury. * Estimate of age based on mean baseline age and time since injury.

A graphical comparison of median KOOS subscales with thresholds is shown in Figure 2. Comparison is also made with published data from the Multicenter Orthopedic Outcomes Network (MOON) cohort [16] to observe any similarities with cohorts at a further time-point from injury (10 years post-ACL).

A total of 153 participants completed at least two testing sessions (*n* = 76 uninjured, *n* = 77 previously injured). The median time between follow-up was 23.4 months (range 8 to 42 months). Reasons for study withdrawal included relocation (16%; 5 cases, 11, controls), not interested (8%; 4 cases, 4 controls), too busy (17%; 9 cases, 9 controls), could not contact (47%; 19 cases, 29 controls), new injury or re-injury (6%; 3 cases, 3 controls), unknown reason (3%; 1 case, 2 controls), pregnancy/recent birth (3%; 1 case, 2 controls) and personal reasons (1%; 1 case). Baseline characteristics for participants who were lost to follow-up are compared with those who returned for follow-up in Appendix A.

The proportion of participants whose KOOS scores were deemed to have “changed” based on MDC criteria are presented in Table 3. To observe clinical features of participants with worse KOOS scores on follow-up, descriptive statistics are presented for injured youth and young adults with at least one KOOS subscale that worsened (*n* = 27) in Table 4. There were nine injured participants who worsened on at least two subscales, four who worsened on at least three and one who worsened on four subscales. Participants worsened on the symptoms’ subscale most commonly (*n* = 18), followed by pain and QOL (*n* = 16 for each), ADL (*n* = 4) and sport and recreation (*n* = 4).

## 4. Discussion

This study applied a number of different criteria to KOOS outcomes to determine a painful or symptomatic knee in the 3–12-year interval following intra-articular knee injury. It is the first study to apply these methods during this interval and across a variety of knee injuries and matched controls, in an effort to characterize the period between injury and potential knee OA development in youth and young adults.

Multivariable logistic regression showed higher odds of having a painful or symptomatic knee for each criteria in both those with an ACL and/or meniscus injury and other sub-groups of injury compared to uninjured controls. The focus of knee injury research and major cohort studies has traditionally been on ACL or meniscus injury due to a high injury burden and significant risk for joint disease. The results of this study suggest that other sub-groups of injury (e.g., collateral ligament and patellar dislocations and subluxations) may carry a high risk for a painful or symptomatic knee requiring medical attention. A lower proportion in this sub-group may have lasting problems across sub-scales: there were 9% (*n* = 3) reporting a symptomatic knee, compared to 18% (*n* = 16) in the ACL/meniscus group. However, of note, 31% (*n* = 11) had significant pain on the PASS criteria compared to 28% (*n* = 25) in the ACL/meniscus group. Since the experience of pain drives healthcare usage and is the top concern of people living with knee OA [17], individuals with other types of knee injury are also worthy of further investigation and research. While it is not known which patients will progress to development of joint disease, it appears as though individuals with traumatic knee injuries that are typically considered “less severe” compared to ACL and meniscus injuries have a similar level of pain and symptoms at 3–12 years following injury.

Sex was not observed to be a significant covariate in the regression models, even though it has been reported that females often report worse knee KOOS outcomes in the first two years following ACL injury [18]. Despite the wide range of post-exposure years included and an expectation that more adverse outcomes consistent with osteoarthritis would be present further from injury, time since injury did not influence the findings. It is worth considering that time since injury may have an impact on the actual thresholds applied since the population assessed is different to the reference populations. Nonetheless, this finding appears to be consistent with other large prospective cohort studies of ACL injury. The MOON cohort reported similar median (IQR) scores for KOOS pain at two years (92 (83–97)), six years (94 (86–100)) and ten years (94 (86–100)) of follow-up [18]. The proportion of individuals in the MOON cohort who satisfied Englund criteria for a symptomatic knee were 43% at two years and 39% at six years [19]. This is higher than the reported 18% of ACL/meniscus injury participants in the current study and is likely because this threshold is primarily based on a low-QOL KOOS sub-scale. As observed in Figure 2, median scores for 10-year data in the MOON cohort show similarities to the PrE-OA cohort for pain, symptoms, ADL and sports/recreation subscales, but are much lower for the QOL sub-scale. It is unknown why this difference between cohorts exists, but could be related to regional differences between populations, healthcare experiences or a younger age (≤18 years) at the time of injury in the PrE-OA cohort in comparison to a baseline median age of 24 years in the MOON cohort. Younger patients have been shown to score higher outcomes post-ACL injury [20].

The development and application of thresholds for PROs is an important step in the clinical identification of patients deemed at risk of adverse symptoms or requiring intervention. However, it is unknown whether thresholds developed on a specific cohort at one point in time can be applied to other cohorts at different time-points from injury. For this reason, three different criteria were applied to the current data, at a median of 7.8 years following knee injury. Across KOOS sub-scales, 59.6% to 96.6% (ACL and/or meniscus) and 68.6% to 100% (other knee injury) had satisfactory scores above the Ingelsrud et al. PASS thresholds established on the NKLR cohort two years after ACL reconstruction. PASS proportions were quite similar using Muller et al. thresholds, with the exception of symptoms, which had a much lower threshold (94.4% to 100%), and ADL, which had a high threshold (48.3% to 65.7%). Although this reference data had a larger follow-up time of 3.4 years (range 1–5 years), these two thresholds did not appear to fit well with the current data or compared to the NKLR cohort. This may be due, in part, to the different analysis approach taken using a receiver operating characteristic (ROC) curve, with lower sensitivity and specificity values reported for symptoms and ADL. One other study [21] applied Muller et al. PASS thresholds to an ACL cohort 10 years following injury in the Swedish National Knee Ligament Register (SNKLR). Proportions of participants reporting satisfactory outcome by sub-scale matched well with the current study, but again were lower on QOL (100% for PrE-OA ACL/meniscus vs. 67.9% for SNKLR), and also sport and recreation (92.1% for PrE-OA ACL/meniscus vs. 57.2% for SNKLR). Similarly, this is likely explained by an older age at time of injury (median 24.8 years).

In order to better understand the time period following knee injury, a longitudinal assessment of PRO’s was explored as a secondary objective. Change in scores was assessed using MDC criteria over a 1–4-year follow-up to identify the extent of outcome decline over a short period. Results were quite variable on different sub-scales and revealed a similar proportion of injured participants that improved (6.5–18.2%) as those that worsened (3.9–16.9%). This may suggest that fluctuations or flare-ups in symptoms are a common feature in this timeframe. Surprisingly, the number of uninjured participants who had an important or detectable improvement (1.3–11.8%) or worsening (1.3–6.6%) on follow-up was higher than expected and underscores the importance of having a control group in these studies. However, the low number of individuals involved warrant some caution regarding interpretation.

Of the injured participants, 35% worsened on at least one KOOS sub-scale in the 1–4-year follow-up. While we did not power the study to detect significant differences on this outcome, observation of baseline characteristics showed that the group who worsened may have been younger, with a higher proportion of ACL and/or meniscus injuries. Mean values for body mass index and fat mass index were higher in those who worsened, consistent with previous evidence of body mass index as a risk factor for worse two-, six- [19] and ten-year outcomes [18]. However, this was unlikely to be a statistically significant difference in these sub-groups. Values for knee extension strength and triple single leg hop appear to be lower at baseline for those with worse KOOS outcomes at follow-up, but this is unlikely to be clinically or statistically significant. Further research is required to confirm this data and may help clinicians to distinguish patients that are likely to require more attention and intervention.

A limitation of this study is that responder criteria and thresholds from an ACL cohort two years post-reconstruction, an ACL cohort one to five years post-reconstruction and a meniscectomy cohort 16 years post-surgery were applied to youth and young adults 3–12 years following knee injury. It is important to consider that thresholds used were not population-specific and this is acknowledged in the interpretation of these findings. Responder criteria thresholds have not previously been applied to injury types other than ACL or meniscus injury. We investigated criteria developed on other cohorts in order to provide comparison across populations and for lack of a more specific option to define post-injury knee problems or early symptomatic OA. An advantage of this approach was the ability to compare published thresholds alongside uninjured matched controls and sub-groups of injury. This evaluation would benefit from replication with a larger number of participants for sub-group analysis. As demonstrated by the wide confidence intervals for multivariable logistic regression, there were likely not enough events per variable which introduced sparse data bias to the analysis. Despite this, a significant association was observed. In addition, numbers were not large enough to statistically account for covariates other than sex and time since injury, such as body or fat mass index, surgery, re-injury or physical activity. Age was not included in models due to collinearity with time since exposure. However, a sensitivity analysis with age included instead of time since exposure did not change the results. Observation of a cohort who were inured under the age of 18 years was a strength of this study since incidence of ACL injury is increasing most rapidly in this age group [22,23], and they may suffer from more long-term health outcomes [24]. Due to the time period under investigation, it was not possible to control for the specific mechanism of injury or type of rehabilitation in the acute injury stage, which may influence the impact on cartilaginous structures, recovery and thus long-term outcomes. Loss to one year of follow-up in this study was 38% for injured participants and 44% for controls, which poses a threat to validity of the longitudinal assessment. Considerable effort was made by the study coordinators to retain participants and withdrawal was mainly due to relocation, university attendance and contact phone numbers that were no longer in use. While the number lost to follow-up is higher compared to other knee injury cohorts, the nature and length of the testing protocol (full procedure included blood draw, biomechanics assessment, clinical exam, shuttle run and range of questionnaires in addition to the above) created a higher participant burden than questionnaires alone. However, age, sex, type of injury and time since injury were very similar for those who were retained vs. lost to follow-up. In an attempt to capture more adverse changes over a longer time period, the time between longitudinal follow-ups was wide-ranging (8 to 42 months). In order to retain numbers for analyses, a more targeted approach (e.g., one-year or two-year change) was not used, but future analysis may benefit from this approach.

## 5. Conclusions

In this younger cohort of individuals with a 3–12-year history of different types of intra-articular knee injury, a high proportion (28–31%) were experiencing significant pain, while 9–18% met the criteria for a symptomatic knee, significant enough to seek medical attention. Odds for significant pain were equally high in those with ACL and/or meniscus injury and other (e.g., collateral ligament) injury compared to controls, demonstrating that a “less severe” injury may also require follow-up and intervention. Over a 1–4-year follow-up, 36% of injured participants worsened on at least one KOOS sub-scale. However, more research is required to validate thresholds specific to populations in the interval between youth knee injury and risk of joint osteoarthritis to accurately observe clinically significant fluctuations in symptoms in this time period. This will aid the development of targeted interventions to decrease the long-term public health burden of youth knee injury.

## Figures and Tables

**Table 1:** Thresholds for The Knee injury and Osteoarthritis Outcome Score (KOOS) corresponding to the patient acceptable symptom state (PASS) and symptomatic knee for cross-sectional data, and minimal detectable change (MDC) for longitudinal change.

Thresholds				
	PASS [10] (Ingelsrud et al.)	PASS [11] (Muller et al.)	Symptomatic Knee [12] ^¥^ (Englund et al.)	MDC [13] (Collins et al.)
Pain	89	88.9	86.1	6.1
Symptoms	83	57.1	85.7	8.5
ADL	95	100	86.8	8.0
Sport/Rec	72	75	85.0	12.0
QOL	73	62.5	87.5	7.2
KOOS 4	79	-	-	-

ADL, activities of daily living; Sport/Rec, sport and recreation; QOL, quality of life. ^¥^ Definition of “symptomatic knee” based on QOL threshold plus at least two other subscale thresholds.

**Table 2:** Descriptive statistics and proportion of participants in the Alberta Youth Prevention of Early Osteoarthritis (PrE-OA) cohort meeting patient acceptable symptom state (PASS) for KOOS outcomes by Ingelsrud [10] and Muller [11] thresholds and proportion passing Englund [12] thresholds.

KOOS	Uninjured (*n* = 129)				ACL and/or Meniscus (*n* = 89)				Other Knee Injury (*n* = 35)			
	Median (IQR)	PASS% Ingelsrud	PASS% Muller	% Englund	Median (IQR)	PASS % Ingelsrud	PASS% Muller	% Englund	Median (IQR)	PASS % Ingelsrud	PASS% Muller	% Englund
Pain	100 (5.6)	91.5	91.5	95.4	94.4 (13.9)	71.9	71.9	80.9	97.2 (11.1)	68.6	68.6	88.6
Sym.	96.4 (10.7)	85.3	100	85.3	85.7 (17.9)	59.6	94.4	59.6	89.3 (10.7)	71.4	100	71.4
ADL	100 (0)	96.1	77.5	99.2	98.5 (4.4)	79.8	48.3	93.3	100 (2.9)	88.6	65.7	94.3
Sport	100 (2.8)	100	99.2	96.9	91.7 (13.9)	95.5	92.1	79.8	97.2 (11.1)	100	100	85.7
QOL	100 (2.8)	100	100	97.7	88.9 (11.1)	96.6	100	61.8	94.4 (5.5)	100	100	85.7
KOOS 4	98.2 (5.1)	98.5	N/A	N/A	90.7 (11.3)	80.9	N/A	N/A	93.7 (6.5)	88.6	N/A	N/A
* Englund	N/A	N/A	N/A	98.5	N/A	N/A	N/A	82.0	N/A	N/A	N/A	91.4

ADL, activities of daily living; IQR, interquartile range; KOOS, knee injury and osteoarthritis outcome score; N/A, not applicable; QOL, quality of life; Sport, sport and recreation; Sym, symptoms. * Proportion that did not have a “symptomatic knee” based on Englund criteria of meeting QOL subscale threshold and at least 2 other subscale thresholds.

**Table 3:** Frequency and proportion (*n* (%)) of uninjured and injured participants with improved or worse KOOS scores by minimal detectable change (MDC) in a 23.4 months (range 8 to 42 months) follow-up.

	Uninjured (*n* = 76)		Previous Injury (*n* = 77)	
	MDC *n* (%)		MDC *n* (%)	
	Improved	Worse	Improved	Worse
Pain	4 (5.3)	5 (6.6)	14 (18.2)	11 (14.3)
Symptoms	9 (11.8)	5 (6.6)	14 (18.2)	13 (16.9)
ADL	1(1.3)	1(1.3)	5 (6.5)	3 (3.9)
Sport/Rec	1(1.3)	1(1.3)	5 (6.5)	3 (3.9)
QOL	6 (7.9)	5 (6.6)	7 (9.1)	11 (14.3)
KOOS 4	-	-	-	-

**Table 4:** Baseline clinical features for injured participants who had worse KOOS scores at follow-up (at least one KOOS subscale worse than the minimal detectable change) and other injured and uninjured participants using mean (95% confidence interval (CI)) or median (25th, 75th centile).

Outcome at Baseline	Injured Participants with Worse KOOS Score(s) at Follow-Up (*n* = 27)	Injured Participants with No Change or Improved KOOS Scores at Follow-Up (*n* = 50)	Uninjured (*n* = 76)
Sex (%)	56% Female	54% Female	53% Female
Age (years) *	22.6 (20.4, 24.5)	23.6 (21.5, 24.9)	23.0 (20.5, 24.5)
Time since injury (years) *	6.0 (4.8, 8.7)	7.0 (5.4, 8.0)	-
Time between tests (m) *	23.6 (18.4, 24.8)	23.4 (17.5, 25.1)	23.3 (14.5, 25.8)
Injury type	82% ACL and/or meniscus 18% Other knee injury	64% ACL and/or meniscus 36% Other knee injury	-
Body mass index (kg/m^2^)	25.4 (24.0, 26.9)	24.8 (23.8, 25.9)	23.4 (22.8, 24.0)
Fat mass index (kg/m^2^)	6.10 (5.09, 7.10)	5.56 (4.84, 6.27)	4.67 (4.24, 5.09)
Knee extension strength (N/m/kg^−1^)	1.77 (1.51, 2.03) ^Δ^	1.89 (1.74, 2.04)	2.00 (1.88, 2.13)
Triple single leg hop (cm)	411.3 (380, 442)	427.6 (405, 451)	443.0 (424.2, 461.7) ^¥^
KOOS			
Pain *	97.2 (91.7, 100)	93.1 (86.1, 97.2)	100 (97.2, 100)
Symptoms *	85.7 (78.6, 96.4)	89.3 (75.0, 96.4)	96.4 (89.3, 100)
ADL *	98.5 (97.1, 100)	98.5 (95.6, 100)	100 (100, 100)
Sport/Rec *	97.2 (88.9, 100)	94.4 (86.1, 97.2)	100 (95.8, 100)
QOL *	91.7 (83.3, 97.2)	91.7 (96.1, 94.4)	100 (97.2, 100)

* Median (IQR), ^Δ^ *n* = 26, ^¥^ *n* = 75.

## Appendix A

**Table A1:** Baseline characteristics of those who completed at least two measurements vs. participants who were lost to follow-up and eligible for study objectives.

Outcome	Completed Follow-Up (*n* = 153)	Lost to Follow-Up (*n* = 100)
Injured/Uninjured (% injured)	77/76 (50% injured)	47/53 (47% injured)
Female/Male (% Female)	82/71 (54% female)	56/44 (56% female)
Age at study entry (years) (Median; 25th, 75th percentile)	23.1 (21.3, 24.5)	22.6 (20.9, 24.5)
Time since injury (years) (Median; 25th, 75th percentile)	6.9 (5.4, 8.3)	6.5 (5.2, 8.2)
Type of injury	70% ACL and/or meniscus 30% Other injury	74% ACL and/or meniscus 26% Other injury
